# Profile, referral pathways and re-attendance of psychiatric patients attending the emergency department: focus on suicidality & self-harm

**DOI:** 10.1192/bjo.2021.890

**Published:** 2021-06-18

**Authors:** Haridha Pandian, Nilamadhab Kar

**Affiliations:** 1 Royal Wolverhampton NHS Foundation Trust; 2 Black Country Healthcare NHS Foundation Trust

## Abstract

**Aims:**

The number of patients presenting to Emergency Departments (EDs) in the UK with acute psychiatric issues is a major concern. This project aimed to explore the outcome of patients assessed by Mental Health Liaison Services (MHLS) in a large district general hospital ED in the UK, with a focus on patients with self-harm or suicidality.

**Method:**

Data were extracted from electronic patient records on 100 consecutive attendees to MHLS in July 2020. Data were collected on demographics, index of multiple deprivation (IMD) by postcode, time and reason for attendance, known ICD-10 diagnoses, self-harm history, alcohol/substance misuse at time of presentation, recent psychosocial stressors and outcome of MHLS assessment. Assessments by MHLS in the preceding 12 months and reattendance to the service within 3 months following this assessment were also recorded.

**Result:**

The sample included 44 male and 56 female patients, with a mean age of 35.3 years. 80.0% of patients were Caucasian. 67.0% lived in areas classed within the top 30% most deprived in the country, whilst 2.0% had no fixed abode. The majority (79.0%) of patients self-presented; outside of normal working hours (70.0%). The most common reasons for attendance were thoughts/intent of self-harm/suicide (50.0%), overdose (29.0%) and self-harm by laceration (7.0%).

The majority (73.0%) of patients had a known psychiatric diagnosis, with the most frequent being depressive disorder (36.0%) and emotionally unstable personality disorder (15.0%). Almost half (48.0%) had a history of self-harm, and 40.0% were under the influence of alcohol/illicit substances upon presentation to ED. The most common psychosocial triggers were conflict with partner (26.0%) and stress due to the COVID-19 pandemic (19.0%).

Following assessment, 24.0% of patients were discharged to their General Practitioner, 18.0% to the community mental health team; and 17.0% to the Crisis & Home Treatment Team. A minority (13.0%) were admitted to a psychiatric hospital (10.0% informally, 3.0% under the Mental Health Act 1983).

Approximately one in five (21.0%) patients re-attended to MHLS within 3 months. Out of 37 patients that had previously been assessed by MHLS in the preceding 12 months; 37.8% were reassessed within 3 months (p < 0.01).

**Conclusion:**

In the studied sample, most (90%) of psychiatric patients attended ED for self-harm or suicide, and a significant proportion had repeat attendance. Socioeconomic deprivation, substance misuse, relationship difficulties and stress due to the COVID-19 pandemic were major issues, alongside diagnosed depression and personality disorder. Focussed support for these issues may decrease ED attendance due to self-harm/suicidality.

